# Does the Attention of the Chinese Government Influence Chinese Nutrition, Exercise, and Health? Based on the Content Analysis of the Central Government Work Reports From 1978 to 2020

**DOI:** 10.3389/fnut.2021.724176

**Published:** 2021-10-18

**Authors:** Wenpeng Zhang, Yuomei Zhou, Jingzhan Li, Tingting Zeng, Jinlian Liao

**Affiliations:** ^1^Department of Research Institute of Healthy China, University of East China Jiaotong University, Nanchang, China; ^2^Department of Physical Education and Health, University of East China Jiaotong University, Nanchang, China

**Keywords:** the Central Government of the People's Republic of China, nutrition, exercise, health, attention, the distribution of resources, government work

## Abstract

The attention of the Chinese government on nutrition, exercise, and health refers to the attention degree of the Central Government to the nutrition, exercise, and health of Chinese nationals and reflects whether Central Government attach importance to Chinese nationals' nutrition, exercise and health or not and the distribution of resources, which influence the physical quality and health level of Chinese nationals. Based on the attention theory and attention distribution proposed by Herbert Simon, Dai Kai, et al., this study took 43 Central Government Work Reports from 1978 to 2020 as research samples, used literature reviews, and textual analysis methods, and applied the Nvivo12.0 software to conduct qualitative and quantitative analyses about the contents of the Central Government Work Report concerning the nutrition, exercise, and health of Chinese nationals. This research found the following: (1) There has been a relatively huge overall change in the attention of the Central Government, that is, the level of attention, to the nutrition, exercise, and health of Chinese nationals from 1978 to 2020, and the policies related to nutrition, exercise and health of Chinese nationals issued by the Central Government have been growing faster. (2) The income level of the urban and rural residents, the total production of various types of food, dietary structure, the total number of medical and health institutions, the average life expectancy of the Chinese population, and the number of sports venues have been constantly increasing since the reform and opening up, which has effectively promoted the improvement of the nutrition, exercise and health level of Chinese nationals, and it cannot be achieved without the attention and support of the Central Government. However, the change in the lifestyle of Chinese nationals has led to the growth of the modern “Civilization Disease,” which is also an important issue that the Central Government needs to handle urgently.

## Introduction

The Outline and Plan of “Healthy China 2030” issued by the Communist Party of China of Central Committee (hereinafter referred to as CPC Central Committee) and the State Council of the People's Republic of China (hereinafter referred to as State Council) that is the Central Government of the People's Republic of China (hereinafter referred to as Central Government) proposed that “health is an inevitable requirement for promoting human beings' all-round development, and the realization of national health and longevity is an important symbol of the country's prosperity, strength, and national rejuvenation” (1). Does the attention degree of the Central Government to the nutrition, exercise, and health of Chinese people influence the health condition of Chinese people? The Central Government Work Report (hereinafter referred to as Government Work Report) refers to the official policy document of the Central Government that summarized the economic and social development of the previous year and set the expected plans and goals for government work in the coming year based on the actual conditions and needs (2), which reflect the attention of the Central Government to various social affairs including nutrition, exercise, and health. Herbert Simon believed that “attention refers to the process in which managers selectively paying attention to certain information while ignoring other parts”(3). However, attention distribution refers to the attention configuration of the action body (4). Based on this, the attention distribution of the government refers to the attention of the Central Government to nutrition, exercise, and health at the same time.

The scope of this research on the government work report from the perspective of attention distribution was wide: vertically, there were both the central level and local level; horizontally, there were the ecological environment level, urban and rural development level, education level, science and technology level, and so on. The researchers analyzed the changed rules of the regional green development based on the government work reports of Beijing, Tianjin, and Hebei province, and provided suggestions for the optimization of the attention level of the regional green development (5). One researcher took 20 provincial policies of eco-environmental protection as the samples to analyze the level, characteristics, and deficiencies of the allocation of the attention of the local government with regards to environmental governance (6). Some researchers took the central policy documents from 1986 to 2019 as the research samples to explore the changing rules of the attention of the Central Government to urban community governance, which provided a reference for promoting urban community governance (7), others analyzed the “*Key Points of Works*” that was issued by the Ministry of Education of the People's Republic of China from 1987 to 2019 through five dimensions, and uncovered the characteristics of the attention distribution of the Central Government to vocational education, and put forward suggestions to optimize the attention distribution levels of the Central Government to vocational education (8). One researcher took 42 Government Work Reports since the reform and opening up as the research samples and found that the attention of the Central Government to scientific and technological innovation has experienced certain periodic changes, and put forward proposals to increase the attention of the Central Government to the field of scientific and technological innovation (9). From the perspective of attention and government decision-making theory, the researchers explored the allocation level of the attention of the Central Government to the cause of aging based on the content analysis of the Government Work Reports from 1978 to 2018, which promoted the development of the cause of aging (10). Whereas, the Chinese researchers who took the Government Work Report as the research samples for analysis found that applied attention theory to nutrition, exercise, and health was rare. Therefore, the research does not only validate the hypothesis that the attention of the Central Government influences Chinese nutrition, exercise, and health, but also fills the gap in the application of attention theory in this field, and promotes the construction of a healthy China.

## Materials and Methods

### Theoretical Foundation

The term “attention” originated from psychology, which refers to the ability of the action body to point and concentrate on something accompanied by a series of psychological processes (11). In the 1940s, Herbert Simon applied “attention” to the field of management, and believed that “attention refers to the process of managers selectively paying attention to some information while ignoring other parts” (3). He also put forward the theory of limited rational decision-making model, which stated that “attention is a kind of scarce resource, and the process of decision-makers' choice is the process of how to effectively distribute limited attention, that is, the process of attention distribution or transfer” (10). In the 1950s, based on the attention distribution proposed by Simon, James March put forward that “attention distribution will be influenced by decision-makers' subjective and objective factors, such as limited rationality, information overload and decision-making environment” (12). In the 1990s, William Ocasio considered that “the decision-making behavior of an organization is built on its attention distribution and influenced by the decision-making environment” (13). Bryan Jones applied “attention” to the field of government decision-making, and believed that “attention is a selection mechanism, all decisions involve selection, and the changes of government policies are constantly varying with the changes of decision-makers' attention” (14).

### Research Methods and Tools

#### Text Analysis

Text analysis is a content analysis method that combines quantitative and qualitative analysis. It was used primarily in the field of informatics and intelligence at the beginning, and then gradually extended to various fields (15). Language usually reflects the cognitive tendency of people, and the cognitive changes of people and the attention degree to things are often reflected in the changes of the frequency of the use of words (12). Therefore, the literal expressions about nutrition, exercise, and health in the Government Work Reports reflected the attention degree of the Central Government to them and their changing process.

#### Use of the Nvivo12.0 Software

In 1999, the American QSR company developed a qualitative analysis software -Nvivo (16), which not only helped researchers organize, analyze, and query unstructured or qualitative data, but also improved the reliability and validity of studies (17). At present, Nvivo12.0 is the latest version. According to the need of the research, we used Nvivo12.0 to code and analyze the text content about nutrition, exercise, and health in the Government Work Reports.

#### Sample Selection

The reasons why the researchers selected 43 Government Work Reports from 1978 to 2020 as the analysis sample were as follows: The first reason was that the Central Government was one of the main management bodies of various social affairs (18), the attention of the Central Government to the nutrition, exercise, and health of Chinese nationals determined the degree of emphasis of the local government to the nutrition, exercise, and health of Chinese nationals, which influenced their physical quality and health level, and the Government Work Report was a real reflection and behavior imprint of the government in dealing with public affairs (19) that was also the representative of the most authoritative, holistic, and prospective (2). The second reason was during “the Ten-Year Cultural Revolution,” the development of various social affairs in China was frustrated, and was gradually recovered and adjusted at the beginning of the reform and opening-up, thus the Government Work Reports since the reform and opening-up were selected to ensure the integrity of the samples.

## Results and Discussion

### The Changes in the Attention of the Central Government Toward the Nutrition, Exercise, and Health of Chinese National Since the Reform and Opening-Up

**Figure 1:**
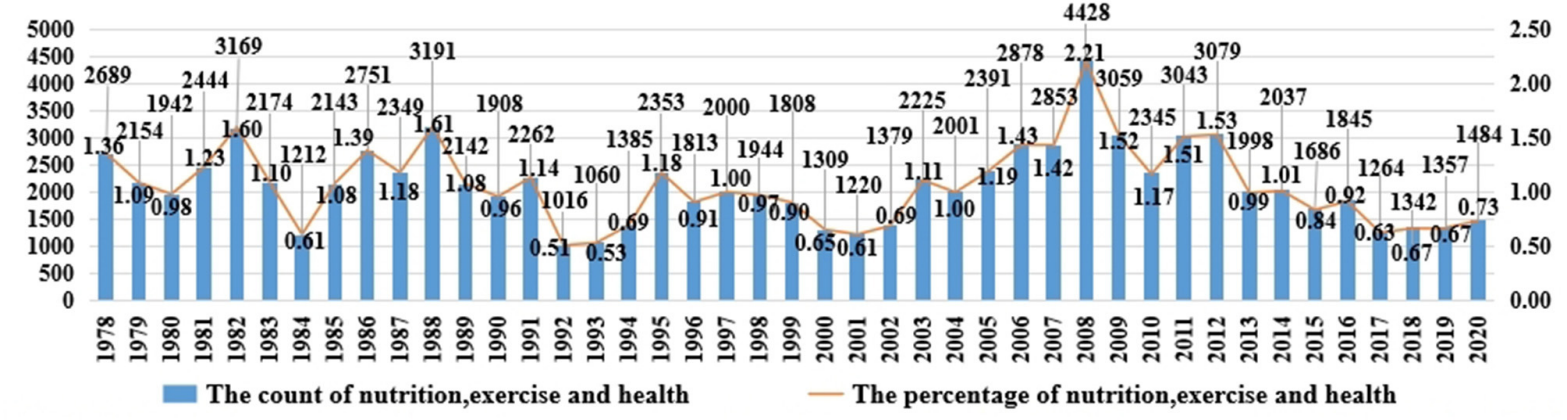
The changes abount the counts and percentages of nutrition, exercise and health in Government Work Report from 1978 to 2020.

The Government Work Report was not only the declaration and programmatic document of CPC Central Committee and State Council on the development of various social affairs (18), but also the baton for the government at all levels to allocate resources and invest energy (2), which implied the intensity of attention and change in the logic of the Party and government on the various social affairs and various fields in China. The “policy discontinuity equilibrium” theory held that “influenced by policy environment and other factors, the policy will experience a long period of stability and sudden change period” (20). Hence, as an official policy document, the Government Work Report over the years would also go through the period. Since the reform and opening up, the discourse expression on the nutrition, exercise, and health of the nationals in the Government Work Report has changed with the policy environment and also reflected the changes in the attention of the Central Government toward the nutrition, exercise, and health of Chinese nationals. After importing the 43 Government Work Reports into Nvivo12.0 and performing a text search query with “nutrition” as the keyword, the research found that there were only five text expressions with “nutrition” as the keyword in Table 1, given that nutrition originated from diet and the sources of diet include agriculture, forestry, animal husbandry, fishery, and so on. Therefore, all the words related to the sources of nutrition in the Government Work Reports over the years were the manifestation of the attention of the Central Government to nutrition. And that exercise and health include physical exercise, sports and fitness, medical care, nutrition, and hygiene (21). Hereby, “nutrition, exercise, and health” was considered as the node, and “nutrition,” “diet,” “food safety,” “agriculture,” “sports,” “fitness,” “medical,” and “hygiene” were used as the keywords to carry out the text search query and code. Drawing lessons from the hypothesis of Zhi Xu, et al., the research regarded the ratio of the number of words related to nutrition, exercise, and health in the Government Work Report to the total number of words in the Government Work Report of that year as the intensity of the attention of the Central Government to the nutrition, exercise, and health of Chinese nationals (2). After the calculation, it was presented in the form of the combination of bar chart and line chart in Figure 1. The research combined the process and staged the characteristics of the Chinese economic, social, and market reform since the reform and opening up with the practice of the nutrition, exercise, and health of the Chinese nationals, and divided the attention of the Central Government to the nutrition, exercise, and health Chinese nationals into three stages: Recovery reform period (from 1978 to1992); Market reform period (from 1993 to 2012); Deepening reform period (from 2013 to 2020).

**Table 1:** The year of the literal expression with the keyword “nutrition” in Government Work Reports from 1978 to 2020.

**Year**	**Reference point**	**Coverage rate**
1999	1	0.01%
2012	1	0.01%
2013	1	0.01%
2014	2	0.02%
2018	1	0.01%

### Recovery Reform Period (From 1978 to 1992)

Bryan Jones believed that “all kinds of factors in the decision-making situation have an effect on decision-making” (14). In different decision-making situations, the Central Government paid different attention levels to the nutrition, exercise, and health of Chinese nationals. Due to the long-term impact of the “cultural revolution,” various fields of Chinese social affairs were at a low ebb with many social problems, such as depressed economic development and low living standards. The convening of the Third Plenary Session of the 11th CPC Central Committee ushered in a new historical period of reform and opening up, and the attention of the Central Government to the nutrition, exercise, and health of Chinese nationals also followed the pace of the reform and opening up into a period of recovery and adjustment. Overall, the intensity of the attention of the Central Government to the nutrition, exercise, and health of Chinese nationals greatly changed during this period. The attention level of the Central Government to the nutrition, exercise, and health of Chinese nationals reached 1.36% in 1978, while the attention level from 1979 to 1981 was lower than that in 1978. The reason was that at the first session of the Fifth National People's Congress, the Government Work Report proposed the general arrangement of “implementing prevention as the main work, extensively launching Patriotic Health Campaigns, and actively carrying out mass sports activities to enhance the physical fitness of Chinese nationals” and the Third Plenary Session of the 11th CPC Central Committee passed the “*Decision on Certain Issues of Accelerating Agricultural Development (draft)”* and “*Regulations on the Work of Rural National Communes (trial draft)”*, which promoted the development of the production undertakings in China, such as agriculture, animal husbandry, forestry, sideline, and fishery. In 1982, the attention level of the Central Government to the nutrition, exercise, and health of Chinese nationals reached 1.6% that because, in January 1982, the CPC Central Committee approved the “*Minutes of the National Rural Work Conference (Released by CPC Central Committee (1982) No.1)”*, and pointed out that “the production team should make plans for the comprehensive development of agriculture, forestry, animal husbandry, sideline, fishery, industry, and commerce in accordance with local conditions, and improve the circulation of rural commodities”. In addition, the Central Patriotic Health Campaign Committee and the Ministry of Health of the People's Republic of China jointly issued the “*Notice on Further Development of Patriotic Health Campaigns and the Construction of Socialist Spiritual Civilization.”* The attention level of the Central Government to the nutrition, exercise, and health of Chinese nationals was as low as.56% in 1984. The reason for this phenomenon was that the CPC Central Committee issued the “*Some Issues of Current Rural Economic Policy (Released by CPC Central Committee (1983) No.1)”* and “*Notice on Rural Work in 1984 (Released by CPC Central Committee (1984) No.1)”* which have effectively promoted the all-round development of agriculture, forestry, animal husbandry, sideline and fishery in China, and the living standards of the urban and rural residents were further improved. As a result, the number of the literal expressions on nutrition in the Government Work Reports have declined in recent years, and the Central Government has laid its main attention on the system reform and opening up. Compared with 1984, the attention level of the Central Government to the nutrition, exercise, and health of Chinese nationals increased from 1985 to 1991 and reached a peak state of 1.61% in 1988. In October 1984, the CPC Central Committee issued the “*Notice on Further Development of Sports,”* which once again stressed that “sports is closely related to Chinese nationals' health, and we must adhere to the policy of combining popularization and improvement, focusing on school sports”. In January 1985, the CPC Central Committee and State Council issued “*Ten Policies on Further Activating the Rural Economy (Released by CPC Central Committee (1985) No.1),”* which was beneficial in improving the tight supply of agricultural products and promoting the rationalization of the rural industrial structure and the diversification of the diet of the urban and rural residents. In April of the same year, the State Council approved the “*Report on Several Issues of Health Work Reform (Released by State Council (1985) NO.62)”* by the Ministry of Health of the People's Republic of China, which unveiled the prolog of health reform and promoted the development of Medical and Health Care in China. In January 1987, the CPC Central Committee issued a notice on “*leading Rural Reform deeper (Released by CPC Central Committee (1987) No.1”)* which was an important reason for the peak of the attention of the Central Government in 1998. In January 1989, the State Council approved and forwarded the “*Opinions on Issues Related to the Expansion of Medical and Health Services”* issued by the Ministry of Health, Ministry of Finance, Ministry of Personnel, State Price Bureau, and State Administration of Taxation of the People's Republic of China. However, the attention level of the Central Government to the nutrition, exercise, and health of Chinese nationals fell to 0.51% in 1992, for the reason that: In the 1990s, the State Council approved the “*Regulations on School Sports Work and Regulations on School Health Work”* that was beneficial to improving the physical health level of the youth, so as to enhance the physical quality and health level of Chinese nationals; In October 1991, the State Council issued a “*Notice on Further Invigorating the Circulation of Agricultural Products”*, which was beneficial to solving the problem of the lag in the circulation of agricultural products and laying the foundation for the urban and rural residents to purchase diversified food, obtain the nutrients that their bodies need, and promote good health. Hence, the level of attention of the Central Government was low in 1992.

### Market Reform Period (From 1993 to 2012)

The convening of the 14th National Congress of the Communist Party of China marked that the reform and opening up of China has entered a new stage, and the goal of establishing a socialist market economic system has been made. In this context, the attention of the Central Government to the nutrition, exercise, and health of Chinese nationals had also changed. Overall, the attention intensity of the Central Government to the nutrition, exercise, and health of Chinese nationals has changed greatly during this period. In 1993, the attention of the Central Government to the nutrition, exercise, and health of Chinese nationals was as low as.56%. The main reason was that the State Council issued the “*Opinions on Deepening the Reform of the Health and Medical System”* in 1992, which proposed to reform the health management system and the price system of Medical and Health Services. In addition, according to the general goal of establishing a socialist market economy system proposed by the 14th National Congress of the Communist Party of China, the State Council issued the “*Notice on Accelerating the Reform of Grain Circulation System”* to promote the reform of the grain circulation system toward commercialization and market-oriented operation in February 1993. In 1995, the attention of the Central Government to the nutrition, exercise, and health of Chinese nationals increased to 1.18%. The main reasons are as follows: In May 1993, State Council issued the “*Outline for the Reform and Development of China's Food Structure in the 1990s (Released by State Council (1993) NO.40),”* which was the first document on food nutrition that was issued by the country since the founding of the People's Republic of China; the State Council issued the “*Regulations on the Administration of Medical Institutions (Released by State Council (1994) No.194)”*; the CPC Central Committee and State Council issued the “*Opinions on Agricultural and Rural Work in 1994 (Released by CPC Central Committee (1994) No.4)”* and “*Opinions on Doing the Work in Agriculture and Rural Work well in 1995 (Released by CPC Central Committee (1995) No.6).”* From 1996 to 1999, the attention of the Central Government to the nutrition, exercise, and health of Chinese nationals varied little and the trend remained relatively stable. The following reasons could explain this phenomenon: Firstly, in June 1995, the “*Outline for the National Fitness Program (Released by State Council (1995) No.14)”* proposed that “by 2010, the Chinese national physique and health level will be comprehensively improved, and National Fitness system with Chinese characteristics will be basically built”, and in August of the same year, the 15th plenary session of the Standing Committee of the 8th National People's Congress adopted the “*Sports Law of the People's Republic of China”*, which clearly put forward the requirement that “sports work should adhere to the development of National Fitness Activities as the basis for the development of mass sports activities to improve the physical fitness of the whole nation”; secondly, in January 1997, the CPC Central Committee and State Council promulgated the “*Decision on Health Reform and Development (Released by CPC Central Committee (1997) No.3)”*, which explicitly proposed the goals and guidelines of health work and the principles that need to be followed; thirdly, in October 1998, the “*Resolution on Several Major Issues in Agriculture and Rural Work”* adopted by the Third Plenary Session of the 15th CPC Central Committee proposed that “we must adhere to market-oriented reforms, steadily develop food production, combine agriculture, forestry, animal husbandry and fishery, and ensure the effective supply of agricultural products”. From 2000 to 2002, the reasons for the low attention level of the Central Government to the nutrition, exercise, and health of Chinese nationals were: the National Sports Work Conference held in Beijing in 1999, which discussed the “*Outline of Sports Reform and Development from 2001 to 2010”*; In December 2000, the “*Outline of Sports Reform and Development from 2001 to 2010”* mentioned that “the main goals of sports reform and development including an obvious increase in the popularity of mass sports, the full realization of the National Fitness Plan, and the effective enhancement of Chinese nationals' physical quality”; In November 2001, the General Office of State Council issued the “*Outline for the Development of Food and Nutrition in China from 2001 to 2010 (Resealed by State Council Office (2001) No.86),”* which pointed out that “entering the new century, accelerating food development, improving food structure, raising the nutritional level of the whole people and improving people's physical health are the urgent needs for the improvement of the national overall quality”; In January 2002, the CPC Central Committee and State Council issued the “*Opinions on Doing the Agricultural and Rural Work Well in 2002 (Released by CPC Central Committee (2002) No.2)”*, which put forward that “by 2010, the rural health service system and the rural cooperative medical system adapted to the requirements of the socialist market economy system will be basically established in national rural areas”. From 2003 to 2012, the attention of the Central Government to the nutrition, exercise, and health of Chinese nationals was high and peaked in 2008 at 2.21%. In July 2002, the CPC Central Committee and State Council issued the *Opinions on Further Strengthening and Improving Sports Work in the New Era* which stated that “carrying out National Fitness Activities and enhancing nationals' physical quality is the fundamental task of sports work”. In October of the same year, the CPC Central Committee and State Council issued the “*Decision on Further Strengthening Rural Health (Released by CPC Central Committee (2002) No.13)*.” As the key point of Chinese health work, rural health work was linked to the development of the rural productivity and influenced the overall physical fitness and health level of Chinese nationals. In 2003, the severe acute respiratory syndrome (SARS) epidemic situation strengthened the construction of the national public health system and increased the investment of medical and health resources. In January 2005, the CPC Central Committee and State Council issued the “*Opinions on Several Policies to Further Strengthen Rural Work and Improve Comprehensive Agricultural Production Capacity (Released by CPC Central Committee (2005) No.1”*, which put forward new requirements for Chinese agricultural and rural work. The reasons for the peak level of the attention of the Central Government to the nutrition, exercise, and health of Chinese nationals in 2008 were that: the CPC Central Committee and State Council promulgated the “*Opinions on Developing Modern Agriculture and Solidly Promoting the Construction of a New Rural (Released by CPC Central Committee (2007) No.1)”* and “*Opinions on Effectively Strengthening Agricultural Infrastructure and Further Promoting Agricultural Development and Increasing Farmers' Income (Released by CPC Central Committee (2008) No.1),”* both of which emphasized the issues about agriculture, rural, and farmers (hereinafter referred to as “Three Rural” issues) and the agricultural product safety question; the CPC Central Committee and State Council issued the “*Opinions on Strengthening Youth Sports and Enhancing Youth Physical Fitness (Released by CPC Central Committee (2007) No.7),”* which promoted the healthy growth of the youth and played a role in pushing the development of national health. In March 2009, the “*Opinions on Deepening the Reform of Medical and Health Care System (Released by CPC Central Committee (2009) No.6)”* was issued, which promoted the development of medical and health care and ensured the health of the urban and rural residents in China. In August of the same year, the release of the “*National Fitness Regulations”* not only promoted the development of national fitness activities but also improved the physical quality and health level of Chinese nationals. In January 2010, the CPC Central Committee and State Council issued the “*Several Opinions on Increasing Efforts to Coordinate Urban and Rural Development and Further Strengthening the Foundation of Agricultural Rural Development (Released by CPC Central Committee (2010) No.1)*,” which put forward “accelerating the construction of the quality and safety supervision system and inspection and detection system for agricultural products, and actively developing pollution-free agricultural products, green food, organic agricultural products.” Therefore, the Central Government has paid high attention to the nutrition, exercise, and health of Chinese nationals since 2003.

### Deepening Reform Period (From 2013 to 2020)

The convening of the 18th National Congress of the Communist Party of China marked a new era of socialism with Chinese characteristics (22). The attention of the Central Government to the nutrition, exercise, and health of Chinese nationals has also entered a new stage of deepening reform, following the developmental needs of the new era. From 2013 to 2020, the number of the literal expressions of the nutrition, exercise, and health of Chinese nationals by the Central Government was lower compared with the previous decade, while the number of central-level documents directly issued by the government has been increasing. According to the developmental needs of the new era, the Central Government has made a new deployment arrangement for the relevant work in the field of nutrition for the nationals. In January 2014, the General Office of the State Council issued the “*Outline for the Development of Food and Nutrition in China from 2014 to 2020 (Resealed by State Council Office (2014) No.3)*,” proposing that “China's food production is not able to meet the nutritional needs yet, the residents are undernourished and surplus coexist, and the lack of nutritional and health knowledge, which must be given great attention”. In October 2016, the CPC Central Committee and State Council issued the “*Outline and Plan of ‘Healthy China 2030'.”* The outline proposed that “we should formulate and implement a national nutrition plan, deeply carry out the research on the evaluation of the nutritional functions of food, comprehensively popularize dietary nutritional knowledge, issue dietary guidelines suitable for the characteristics of different groups of people, guide residents to form scientific dietary habits, and promote the construction of a healthy diet culture”. In July 2017, the General Office of the State Council issued the “*National Nutrition Plan from 2017 to 2030 (Resealed by State Council Office (2017) No.60)*,” which put forward that “nutrition is an important material basis for human beings to maintain life, growth, and health, and nationals' nutrition is related to the improvement of nationals' quality and economic and social development.” Since the 18th National Congress of the Communist Party of China, the Central Government has also made a series of new deployment arrangements for the work of national fitness. In October 2014, the State Council issued “*Several Opinions on Accelerating the Development of the Sports Industry And Promoting Sports Consumption (Resealed by State Council (2014) No.46”)* that put forward “promoting National Fitness as the national strategy of China”, which marked a significant leap in the concept and practice of mass sports development in China, recognizing national fitness as the rightful meaning of national health. National fitness is a significant means of national health, it is inevitable to promote the development of national health. In June 2016, the State Council issued the “*National Fitness Plan from 2016 to 2020 (Resealed by State Council (2016) No.37).”* In October of the same year, the “*Outline and Plan of ‘Healthy China 2030”'* was issued by the CPC Central Committee and State Council which was a program of action to promote a healthy China, and “Co-construction and sharing, national health” was the strategic theme of building a healthy China. To accelerate the construction of sports power and to vigorously promote the in-depth integration of national fitness and national health, the General Office of the State Council issued the “*Notice on Printing and Distributing the Outline of Building Sports Power (Resealed by State Council Office (2019) No.40”* in September 2019. In September of the same year, for the sake of actively implementing the national fitness initiative and making regular participation in physical exercise a way of life, the General Office of the State Council issued the “*Opinions on Promoting National Fitness and Sports Consumption and Pushing the High-Quality Development of the Sports Industry (Resealed by State Council Office (2019) No.43).”* In October 2020, the General Office of the State Council issued the “*Opinions on Strengthening the Construction of National Fitness Facilities and Develop Mass Sports (Resealed by State Council Office (2020) No.36)”* to boost the construction of fitness facilities, promote the vigorous development of mass sports, and enhance the level of public services for national fitness. The Central Government has also carried out a comprehensive reform in the development of the medical and health services in China. In September 2013, the State Council issued “*Several Opinions on Promoting the Development of Health Service Industry (Resealed by State Council (2013) No.40)*,” which proposed that “accelerating the development of the health service industry is an inevitable requirement for deepening medical reform, improving Chinese nationals' livelihood and enhancing the health quality of the whole Chinese.” In March 2015, the General Office of the State Council issued the “*Notice on Printing and Distributing the Outline of the National Medical and Health Service System Planning from 2015 to 2020 (Resealed by State Council Office (2015) No.14)”* to promote the further optimal allocation of the Chinese medical and health resources and build an integrated medical and health service system. In August 2018, the General Office of the State Council issued the “*Notice on Printing and Distributing the Reform Plan for the Division of Financial Affairs Powers and Expenditure Responsibilities between the Central and Local Governments in the Field of Medical and Health (Resealed by State Council Office (2018) No.67)*.” In June 2019, the General Office of the State Council issued the “*Notice on Printing and Distributing the Key Work Tasks for Deepening the Reform of the Medical and Health System in 2019 (Resealed by State Council Office (2019) No.28)*,” proposing that “putting Chinese national health at the center, implementing prevention as the main work, strengthening disease prevention and health promotion, deepening the linkage reform of medical care, medical insurance and medicine, and firmly promoting the reform of the Medical and Health System effectively implemented and benefiting the Mass”. In July 2020, the General Office of the State Council issued the “*Key Work Tasks for Deepening the Reform of the Medical and Health System in the Second Half of 2020 (Resealed by State Council Office (2020) No.25)*,” which put forward “strengthening the construction of the public health system and further implement the Healthy China Initiative”.

From the above, it can be seen that since the new era, although the Central Government has paid less attention to the nutrition, exercise, and health of Chinese nationals compared with the previous decade, there has been an increasing number of policies at the central level related to nutrition, exercise, and health after entering the new era, most of which were macro-policies and planning programs. On one hand, the promulgation of these policies showed that the CPC Central Committee and the State Council have attached great importance to the nutrition, exercise, and health of Chinese nationals, and to a certain extent promoted the improvement of national nutrition, exercise, and health in China. On the other hand, it indicated the lack of micro and detailed measures on nutrition, exercise, and health. The nutrition policy and action plan of China have promoted the overall nutritional status of Chinese residents to some extent, but there are still some deficiencies that need to be improved. For example, the lack of laws and regulations on nutrition policy protection, lack of long-term mechanism and sustainability, inadequate nutrition policy system, and so on (23). In addition, studies have shown that the elderly sports policy system in China was not sound (24), public sports health policy was still in the stage of continuous development, and the public sports policy of the scientific and standardized aspects still needed to be improved (25), the school sports policy toolbox was not sound and the policy system was not perfect (26), the youth health policy still faced the problems of vague implementation standards and the imperfect linkage mechanism between the executive departments (27).

### The Changes in the Nutrition, Exercise, and Health Level of Chinese Nationals Since the Reform and Opening-Up

**Figure 2:**
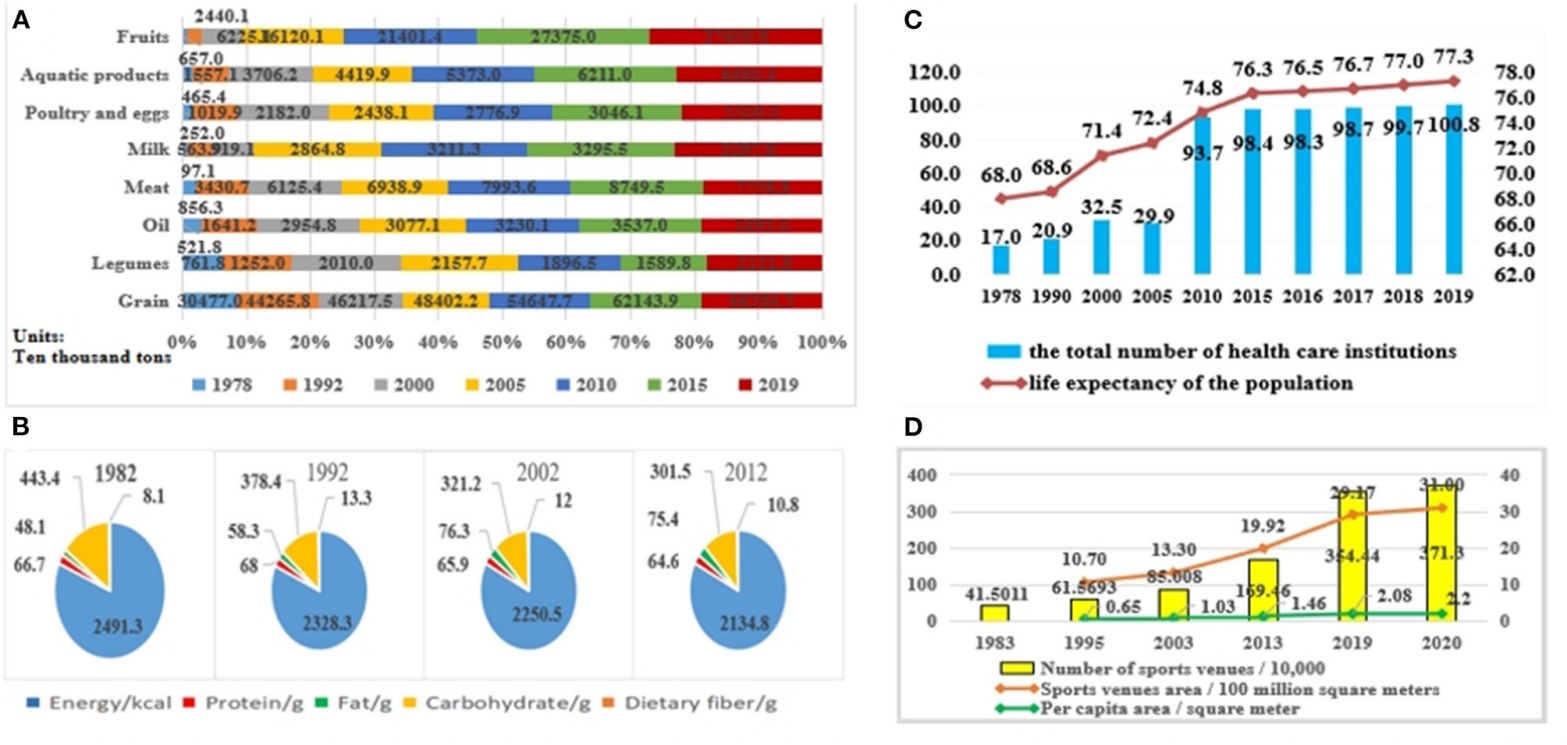
**(A)** Changes in the total production of various types food in China since reform and opening up (Source: Chinese National Bureau of Statistics). **(B)** The energy and the average intake of major nutrients of Chinese residents from 1982 to 2012 (g/standard person day) (Source: China Health Statistics Yearbook). **(C)** Changes in the Average life expectance of the Chinese population and the total number of Medical and Health institutions since reform and opening up (Source: Chinese National Bureau of statistics). **(D)** Changes in the number, area and per capita area of sports venue in China since reform and opening up (Source: Chinese General Administration of Sport).

Since the reform and opening up, the attention of the Central Government to the nutrition, exercise, and health of Chinese nationals has changed significantly. The number of policies related to nutrition, exercise, and health issued by the Central Government has increased, and the level of nutrition, exercise, and health of Chinese nationals has also changed constantly. The per capita income of the Chinese urban and rural residents has been increasing since the reform and opening up. As shown in Table 2, the per capita income of rural residents has increased from 134 yuan in 1978 to 17,131 yuan in 2020, and that of urban residents has increased from 343 yuan in 1978 to 43,834 yuan in 2020. Since the reform and opening up, with the improvement of the per capita income of the urban and rural residents in China and the solving of the problem of food and clothing, the demand of the nation for diet has gradually shifted from being full to eating well, and the dietary structure has gradually developed toward a nutritional and scientific direction (28). The dietary structure refers to the amount of all kinds of food in the daily diet of people and their proportion in the diet (29). There are seven main kinds of nutrients in the human diet, namely protein, fat, carbohydrate, vitamin, mineral (including trace element), water, and fiber (30). As shown in Figure 2A, the changes in the total production of various types of food in China since the reform and opening up reflected the changes in the overall national diet level, which further reflected the changes in the overall national nutritional level. The total national grain production in China rose from 304.77 million tons in 1978 to 663.843 million tons in 2019. Legumes, which are rich in proteins, fats, carbohydrates, and other nutrients, are part of the daily diet of Chinese residents. The production of legumes in China was low in 1978 with only 7.6108 million tons. After the establishment of the socialist market economic reform goal, the production of legumes considerably increased, reaching 21.5767 million tons in 2005. The total production of legumes in China has declined due to the global economic crisis. In 2010, the Chinese legumes production fell to 18.7184 million tons. The Chinese legumes production reached 21.139 million tons in 2019. The Chinese oil production rose from 5.218 million tons in 1978 to 34.93 million tons in 2019. The total production of meat, milk, poultry and eggs, aquatic products, and fruits in China has increased relatively. The increase in the total production of these food items was not only due to the changes in the attention level of the Central Government in the area of nutrition, but also the issuance of policy documents related to nutrition, diet, agriculture, etc.

**Table 2:** Changes in the per capita net income of rural residents and per capita disposable income of urban residents in China since reform and opening up (Source: Chinese National Bureau of Statistics).

**Years**	**Per capita net income of rural residents**	**Per capita disposable income of urban residents**
1978	134	343
1980	191	478
1990	686	1,510
1992	784	2,027
2000	2,253	6,280
2010	5,951	19,109
2020	17,131	43,834

China respectively conducted five national nutrition surveys in 1959, 1982, 1992, 2002, and 2012, and the researchers analyzed the changes in the dietary structure of Chinese residents by using the data of national nutrition surveys comprehensively since the reform and opening up. As seen in Table 3; Figure 2B, the dietary structure of Chinese residents has undergone major changes. Before the reform and opening up, the dietary structure of Chinese residents was categorized under “high carbohydrate, low protein, low fat, low vitamin pattern”. Since the reform and opening up, the dietary structure of Chinese residents has gradually shifted to the “affluent type” pattern of “high energy, high fat, high protein and low dietary fiber” (31). From 1982 to 1992, the average daily intakes of other cereals and tubers decreased significantly, while the average daily intakes of flour and its products, light-colored vegetables, and legumes decreased slightly. On the contrary, the average daily intake of dark-colored vegetables, fish and shrimp, and vegetable oils increased, and the average daily intake of fruits, livestock and poultry meats, rice and its products, eggs and its products, milk and its products, and salt increased slightly. In addition, the average daily intake of protein, fat, and dietary fiber of Chinese residents increased, while the average daily intake of carbohydrates decreased sharply. The average daily intake of energy of Chinese residents also decreased. To effectively avoid the formation of the “affluent type” dietary pattern of “high energy, high fat, high protein, and low dietary fiber”, the first policy document on Chinese national dietary structure was issued- “*Outline for the Reform and Development of China's Food Structure in the 1990s (Released by State Council (1993) NO.40).”* From the early 1990s to the beginning of 2000s, the average daily intake of other cereals, fruits, and salt declined slightly, while the average daily intake of tubers, flour and its products, dark-colored vegetables, and light-colored vegetables decreased sharply. The average daily intake of rice and its products, milk and its products, livestock and poultry meats, and vegetable oils increased significantly, while the average daily intake of legumes, eggs and its products, fish, and shrimp, and animal oils increased slightly. In addition, the average daily intake of Chinese residents when it comes to energy, protein, carbohydrates, and dietary fiber declined, while the average daily intake of fat rose. After entering the 21st century, to promote the development of the Chinese national dietary structure into a nutritious and scientific way, the General Office of the State Council issued the “*Notice on Printing and Distributing the Outline for Chinese Food and Nutrition Development from 2001 to 2010 (Resealed by State Council Office (2001) No.86).”* From 2002 to 2012, the average daily intake of other cereals, tubers, rice and its products, and legumes decreased. Meanwhile, the average daily intake of dark-colored vegetables, light-colored vegetables, fruits, fish and shrimp, milk and its products, animal oils, and salt decreased slightly. On the contrary, the average daily intake of flour and its products, eggs and its products, livestock and poultry meats, and vegetable oils all increased. Moreover, the average daily intake of energy, protein, fat, carbohydrates, and dietary fibers of Chinese residents decreased. To address the coexistence of under-nutrition and over-nutrition and to optimize the dietary structure of Chinese residents, in January 2014, the General Office of the State Council issued the “*Outline for the Development of Food and Nutrition in China (2014–2020)(Resealed by State Council Office (2004) No.3).”*

**Table 3:**
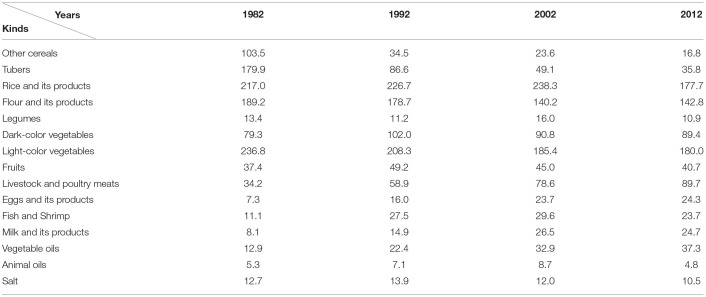
Changes in the average food intake of Chinese residents from 1982 to 2012 (g/standard person day) (Source: China Health Statistics Yearbook).

According to the above, we can know that the relationship between the nutrition level and income level of Chinese residents is not always positive. The increase of the per capita income of Chinese residents to a certain extent contributes to the improvement of the nutritional level of the residents, and the diversification of food also contributes to the diversification of the dietary choices of the residents, similarly, promoting the optimization of the dietary structure of the residents, to some extent, may promote the health level of Chinese residents. However, China has a large population base, and there is still a certain gap in the per capita income between the urban and rural residents. Therefore, the eating habits and levels of Chinese nationals are also different. Eating in restaurants, ordering takeout, and other consumer behaviors, bento, fast food, and other eating habits will lower the health level of Chinese people, followed by the modern “Civilization Disease”. The modern “Civilization Disease” refers to the disease caused by the bad modern lifestyle, including obesity, hypertension, diabetes, and so on (32). In China, six national health service surveys were conducted in 1993, 1998, 2003, 2008, 2013, and 2018, respectively. The results showed that the number of people suffering from chronic diseases in China increased to 204 million (33, 34). Compared with the results of the first National Health Service survey in 1993, the prevalence of circulatory diseases increased dramatically. In the urban areas, the prevalence of circulatory diseases rose from third to second place, with an increase of nearly half (47.1%), while in the rural areas, the prevalence of such diseases increased by 65.57%, although the ranking remained unchanged. Compared to 1998, the main diseases that increased the prevalence of chronic diseases in the urban areas were hypertension (up to 71%), diabetes (up to 120%), and cerebrovascular diseases (up to 33%). The main diseases that increased the prevalence of chronic diseases in the rural areas were hypertension (up to 134%), gallstones and cholecystitis (up to 38%), and cerebrovascular diseases (up to 47%). In 2008, the prevalence of hypertension diagnosed by doctors increased rapidly by 2.2 times in 15 years, and the prevalence of diabetes increased by 2.8 times in 15 years, and the survey estimated that the total number of chronic disease cases reached 270 million, an increase of 0.7 million from 2003. In 2013, the prevalence of self-reported hypertension was 14.2% among the surveyed population aged 15 years and above which increased by 330.3% in 10 years. The prevalence of self-reported diabetes among those aged 15 years and above was 3.5%, which increased four times in 10 years. The prevalence of overweight and obesity among the population aged 18 years and above accounted for 24.8 and 5.4%, respectively. In 2018, the sixth National Health Service Statistics survey report put forward that the increasing chronic diseases were one of the important problems in the healthy development of China in the future (35). Therefore, although the increase of the income of Chinese residents will improve their health level to a certain extent, the interaction of various factors such as the improvement of the technology in China, the enhancement of industrialization, the popularization of electronic products, the acceleration of social pace, and the increase of sedentary behavior has resulted in the lack of physical activity of Chinese people and further led to the increasing rates of obesity, diabetes, cardiovascular and other modern “Civilization Diseases”. In response to the growth of modern “Civilization Diseases”, the General Office of the State Council issued the “*Notice on Printing and Distributing China's Mid-and Long-term Plan for the Prevention and Treatment of Chronic Diseases (Released by State Council Office (2017) No.12)”* in February 2017, which mentioned, “strengthening health education and improving the health quality of the whole people, implementing early diagnosis and treatment to reduce the risk of disease among high-risk groups, promoting the coordination of medical treatment and prevention, realizing the whole process of health management and other measures” (36). In September 2019, Premier Li Keqiang presided over the opening of the China State Council executive meeting, proposing that “the reimbursement rate for over 300 million patients with hypertension and diabetes should be raised to more than 50%” (37). The Customs Tariff Commission of the State Council issued the “*Notice on the adjustment plan of temporary import tariff rate in 2020 (Released by State Council Customs Tariff Commission (2019) No.50)*,” which put stated “implementing zero tariff on several new diabetes drugs.” (38).

Besides the income of urban and rural residents, dietary structure, and lifestyle, many other factors are affecting the health of Chinese nationals, such as health care, sports, and fitness. Medical and health care are related to the health of hundreds of millions of people. Since the reform and opening up, the development level of medical and health care in China has gradually increased, and the average life expectancy of the Chinese population has also risen. As can be seen in Figure 2C, the total number of medical and health institutions in China was 170,000 in 1978. After the establishment of the socialist market economic reform goal, the total number of medical and health institutions in China inflated to around 300,000. Until 2010, the total number of medical and health institutions in China reached 937,000. The main reason for the sharp increase in the total number of medical and health institutions was the “*Opinions on Deepening the Reform of the Medical and Health System (Released by CPC Central Committee (2009) No.6)”* issued by the Central Government and State Council in March 2009, which has not only promoted the development of medical and health undertakings in China but also laid a foundation for the improvement of the whole national health. In addition, the average life expectancy of the Chinese population has increased from 68 years old in 1978 to 77.3 in 2019. The thought of “exercise is a valuable medicine” has existed since ancient times. The leaders of the past generations of the Chinese nation have attached great importance to the physical quality and health level of Chinese nationals and emphasized the importance of exercise and fitness. In June 1952, Chairman Mao Zedong wrote an inscription for the foundation of the All-China Sports Federation which stated to “develop sports and build up the people's physical fitness.” In August 1997, Chairman Jiang Zemin wrote an inscription for the national fitness work which stated that “national Fitness benefits the country and Chinese nationals and contributes to the present and future generations”. In October 2005, when General Secretary Hu Jintao attended the 10th National Games in Nanjing, he proposed that “carrying out national physical fitness activities to improve the health quality of the whole nation.” In August 2013, when meeting with the national mass sports advanced units and advanced individual representatives, General Secretary Xi Jinping emphasized that “national fitness is the foundation and guarantee for all people to enhance their physical fitness and lead a healthy life, national fitness is an important connotation of building moderately prosperous society in all respects, and it's also a vital basis for every person to grow up and live a happy life”. It can be seen from Figure 2D that since the reform and opening up, the number of sports venues in China has increased from 415,000 in 1983 to 3,713,000 in 2020; the area of sports venues has expanded from 1.07 billion square meters in 1995 to 3.1 billion square meters in 2020; the per capita area of sports has increased from.65 square meters to 2.2 square meters. The increase in the number and the area of sports venues can effectively solve the problem of insufficient venues, thereby promoting the increase of the physical exercise population and further improving the physical fitness of Chinese nationals.

## Conclusion

In conclusion, there has been a relatively huge overall change in the attention of the Central Government, that is, the level of attention, to the nutrition, exercise, and health of Chinese nationals from 1978 to 2020, and the policies related to the nutrition, exercise, and health of Chinese nationals issued by the Central Government have been growing faster. The income level of the urban and rural residents, the total production of various types of food, dietary structure, the total number of medical and health institutions, the average life expectancy of the Chinese population, and the number of sports venues have been constantly increasing since the reform and opening up, which has effectively promoted the improvement of the nutrition, exercise, and health level of Chinese nationals, and it cannot be achieved without the attention and support of the Central Government. However, the change of the lifestyle of Chinese nationals has led to the growth of the modern “Civilization Disease,” which is also an important issue that the Central Government needs to handle urgently. Therefore, the research can not only offer a window into the changes of the attention of the Central Government to the nutrition, exercise, and health of Chinese nationals since the reform and opening up, but also verify the hypothesis that the attention of the government influences the level of the nutrition, exercise, and health of Chinese nationals. Furthermore, the research also summarizes the progress and defects of the development in nutrition, exercise, and health in China to lay a good foundation for the promotion of a healthy China.

## Data Availability Statement

The original contributions presented in the study are included in the article/supplementary material, further inquiries can be directed to the corresponding author/s.

## Author Contributions

TZ and JL: data analysis and review and editing. JL: methodology. YZ: check the data. WZ and YZ: draft the paper. All authors have read and agreed to the published version of the manuscript.

## Funding

This research was funded by the National Social Science Foundation of China (Grant Number: 17BTY077) and the Chinese Postdoctoral Science Foundation (Grant Number: 2016M591116).

## Conflict of Interest

The authors declare that the research was conducted in the absence of any commercial or financial relationships that could be construed as a potential conflict of interest.

## Publisher's Note

All claims expressed in this article are solely those of the authors and do not necessarily represent those of their affiliated organizations, or those of the publisher, the editors and the reviewers. Any product that may be evaluated in this article, or claim that may be made by its manufacturer, is not guaranteed or endorsed by the publisher.
